# Swedish Consumers’ Perception of Food Quality and Sustainability in Relation to Organic Food Production

**DOI:** 10.3390/foods7040054

**Published:** 2018-04-01

**Authors:** Techane Bosona, Girma Gebresenbet

**Affiliations:** Department of Energy and Technology, Swedish University of Agricultural Sciences, P.O. Box 75651 Uppsala, Sweden; girma.gebresenbet@slu.se

**Keywords:** organic food, food quality, consumers’ food buying decision, sustainable food production

## Abstract

Consumers’ demand for locally produced and organic foods has increased in Sweden. This paper presents the results obtained from the analysis of data acquired from 100 consumers in Sweden who participated in an online survey during March to June 2016. The objective was to identify consumers’ demand in relation to organic food and sustainable food production, and to understand how the consumers evaluate food quality and make buying decisions. Qualitative descriptions, descriptive statistics and Pearson’s Chi-square test (with alpha value of *p* < 0.05 as level of significance), and Pearson’s correlation coefficient were used for analysis. About 72% of participants have the perception that organic food production method is more sustainable than conventional methods. Female consumers have more positive attitudes than men towards organic food. However, age difference, household size and income level do not significantly influence the consumers’ perception of sustainable food production concepts. Regionality, sustainable methods of production and organic production are the most important parameters to characterize the food as high quality and make buying decisions. On the other hand, product uniformity, appearance, and price were found to be relatively less important parameters. Food buying decisions and food quality were found to be highly related with Pearson’s correlation coefficient of *r* = 0.99.

## 1. Introduction

In Europe, consumers often associate locally produced and organic food products with higher quality standards (freshness, nutritional value), healthy eating, good taste, cultural values, and more environmentally friendly production methods [1,2]. With the increase of consumers’ demand for local produce and organic food in Europe [3], the land covered by organic farm increased from 7.27 million hectares in 2006 to 11.63 million hectares in 2014 [4]. Recent estimates indicate that organic agricultural land covers about 43.7 million hectares of land worldwide, and about 26.6% of this is in Europe [4]. Only in one year (from 2013 to 2014) did the organic agricultural land increase by 2.3%.

According to European Rural Review [3], typical organic farming practices include “multiannual crop rotation; efficient use of on-site resources; strict limits on the use of synthetic pesticides and fertilizers, livestock antibiotics, food additives and processing aids and other inputs; use of plant and animal species that are resistant to disease and adapted to local conditions; and an absolute prohibition of the use of genetically modified organisms”.

According to Regulation (EC) 834/2007 of the European Commission, the overall principles of organic food production include appropriate design and management of biological processes based on ecological systems using natural resources which are internal to the system; restriction of the use of external inputs; strict limitation of the use of chemically synthesized inputs; adaptation of the rules of organic production taking account of sanitary status, regional differences in climate and local conditions, stages of development and specific husbandry practices [5].

Conceptually, sustainability comprises environmental, economic, and social aspects. There is a challenge to have a sustainable food supply due to the increasing world population, urbanization, depletion of resources, as well as spatial and temporal fluctuation in food availability. Therefore, it is important to increase the awareness of consumers of sustainable food production and supply systems. In general, sustainability in the context of sustainable development is defined by the World Commission on Environment and Development [6] as ‘forms of progress that meet the needs of the present without compromising the ability of future generations to meet their needs’.

As a part of sustainable development in the agriculture sector, there is integrated farming, which is a method between conventional and organic farming methods. It reinforces the positive influences of agricultural production and reduces negative impacts [7]. ’Integrated farming makes a vital contribution to sustainable development by adding consideration of economic, ecological and social objectives to the essential business of agricultural food production’ [7].

In Sweden, there are requirements for organic farming which are mainly issued by an association known as KRAV. KRAV is an incorporated association with stakeholders representing farmers, processors, consumer, and firms with environmental and animal welfare interests [8,9]. KRAV is Sweden’s most well-known environmental label for food and beverages, based on ecological principles with especially high standards for animal welfare, health, social responsibility and climate impact. All KRAV-certified operations have to comply with national laws such as animal welfare and environmental legislation. KRAV standards meet other standards of organic production at European and international levels. For instance, KRAV meets standards in IFOAM (International Federation of Organic Agriculture Movements) and standards on organic food at the European level such as Regulation (EC) 834/2007, Regulation (EC) 889/2008, Regulation (EC) 1235/2008 [5,10].

**Figure 1:**
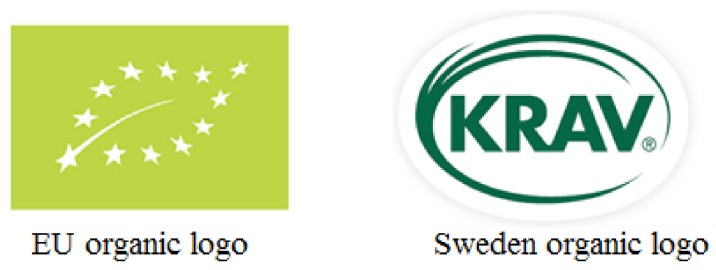
Examples of logos that can be used for organic products.

In the food sector, an organic label is an indication that the food is produced using organic production methods. The European Union (EU) organic logo can be used together with national or private logos. For instance, Figure 1 presents the EU organic logo and the Sweden organic logo that can be used for organic food products.

The positive attitude towards KRAV has increased more in recent years due to the characteristics such as reliability, expertise, high status and modernity associated with KRAV and, currently, more than 98% Swedes are familiar with KRAV [8]. This increased attitude towards the KRAV label indicates that many consumers are deciding to purchase more organic food products, because the decision to purchase a product or service depends on consumer behavior [11]. Although demand for local produces increases and local and organic food sector continues to flourish, further development of the sector still needs effective support [12]. For instance, many tomato growers in southern Sweden perceive that organic farming is effective, but they need strong support and they are willing to shift to organic farming if adequate technical and professionals support is provided [13].

Studying the role of customers’ attitudes to a relationship between environmental knowledge and purchase intention for environmentally sustainable products, Kumar et al. [14] highlighted that attitudes towards environmentally sustainable products mediate the relationship between environmental knowledge and purchase intention. In the food sector, retailers have the potential to promote the purchase and consumption of environmentally more sustainable products [15]. In this regard, organic food store brands have contributed to the development of demand for organic products through supermarkets [16].

In general, consumers tend to buy food products with good taste and price, that are easily available and convenient to purchase as well as environmental friendly. Although about two out of three Swedes tend to buy environmentally friendly products, more than 90 percent of consumers consider that organic products are expensive [17]. In addition to reducing the price difference between organic and conventional products, increasing the awareness of consumers about the benefits of organic products is essential to promote the organic product market. Organic agriculture is a process-oriented rather than a product-oriented production system. Many consumers might find it difficult to understand this concept.

Consumer demand for organic food products in Sweden is increasing sharply. For instance, only in 2015, about 1600 new KRAV-labeled items entered the Swedish market and the market for organic food increased by 39% in the same year [5]. This growth could have been more if it had not been hampered by shortage of raw materials supply. Supplying adequate organic raw materials to the food industry is becoming a challenge in Sweden. Another challenge noticed is the risk of fraud with KRAV label, indicating the need for strong supervision of the system.

In relation to organic food consumption and sustainability issues, there is the consumer group LOHAS (Lifestyle of Health and Sustainability) in Sweden which has a strong positive attitude towards KRAV. Understanding the characteristics of these consumers enables us to understand the expectations of Swedish organic food consumers. The size of LOHAS-consumers interested in organic food (and often willing to pay more for organic food) has increased from 27% of Swedes in 2005 to 38% in 2015 [8]. A socio-demographic based assessment of LOHAS [8] also indicated that typical LOHAS consumers are characterized as women between 35 and 60 years of age, have an above-average income, and have a higher level of education than Swedish people in general. LOHAS is becoming a trend in mostly western countries and is spreading to other parts of world, with about 100 million consumers worldwide [18]. In Europe, the LOHAS consumers group makes up approximately 20% of the European population [18]. This indicates that the LOHAS consumer group could have an influence on the future development of organic food production in Europe and the rest of the world.

Prior to buying a product or service, a consumer searches for information relevant to making a purchase decision [11]. Different sources of information such as the Web, coworkers, and consumer magazines can be used for this purpose. New innovations in information technology and means of communication contribute to the increasing scope of marketing organic food. In this regard, food companies dealing with organic food items indicate that communication with consumers is important, and currently they use different means including social media such as Facebook, LinkedIn, Twitter, Instagram etc. These forums are especially important when launching new KRAV-certified products in Sweden.

The study by Irandoust [19] on Swedish consumers pointed out some factors on which consumer’s choice for organic food depends: perceived benefits of organic food in terms of environment, health, and quality; consumer’s perception and attitudes towards labelling system, message framing, and geographical origin of organic produce; high willingness to pay more for organic food; and income level of consumers. Although consumers’ demand for organic food is increasing, there is less knowledge how the Swedish consumers perceive organic food from sustainable food production and food quality point of view.

The main objective of the current study was to analyze the perception of consumers in Sweden of sustainable food production in relation to organic food production and consumption, food quality as well as food purchasing decisions. This enables us to increase the understanding about consumers’ attitudes towards organic food and sustainable food production discourse. It supports the identification of the roles consumers can play to promote sustainable food supply chains with improved quality, availability, and safety of organic food in particular and all food items in general.

## 2. Methodology

### 2.1. Data Survey

The study is based on literature and data survey on Swedish consumers. About 96% of the data was acquired through online data survey, while the remaining 4% was complemented by retailer-based survey. Online data survey was done in 2016, during March–June. Firstly, retail-gate data collection was planned. Later on, it was understood that online data survey enables us to find more data and a random distribution of customers. The online survey was open for all customers in Sweden, while the retail-gate survey has limitations in covering wider areas. During the survey, consumers were asked to answer 15 questions which were prepared in the Swedish language. Swedish–English language translation was checked by a native Swedish-speaking researcher. The survey was used to gather information on the demographic characteristics of organic food consumers in Sweden. These include gender, age, education status, household income, number of family members, number of children in household, and occupation. Both the online survey and retail-gate survey were open to every interested consumer and did not target only organic food consumers. In general, 107 consumers responded, out of which 100 responses were complete and used in this analysis. The method used is mainly descriptive statistics and qualitative description. Descriptive statistics and Pearson’s Chi-square test were used as statistical tools. The Pearson’s Chi-square test was used to test how responses of consumers differ for specific question under consideration. It is used to test the relationship between variables. In addition, Pearson’s correlation coefficient was used to test the linear dependence between two important food characterizing parameters i.e., consumers’ food quality characterization and their food buying decision.

### 2.2. Hypothesis Based Analysis

The study aimed to investigate how consumers perceive sustainable and organic food production concepts, evaluate food quality and make food buying decision. Based on this, the following seven hypotheses were set and analyzed in order to understand the relation between different factors (see Table 1). In all cases of the null hypothesis (Ho), Pearson’s Chi-square test has been constructed so that the variables under comparison are independent. This was conducted with significance level *p* < 0.05. The tests are done in order to understand how consumers’ gender difference, age difference, education level, household size, and income level influence the perception their attitude towards sustainable food production methods. Similarly, it was intended to highlight how the difference in income level can influence the consumers’ judgment of food quality as well as their food buying decisions.

## 3. Results and Discussion

### 3.1. Demographic Characteristics of Organic Food Consumers in Sweden

The data acquired from data survey is summarized in Table 2. It was found that females, more educated people, and families with higher income level mostly participated. About 61% of the participants were female. The participants were grouped into six groups according to their age, and the dominating age group was found to be 35–44 years old (32% of total) followed by 45–54 years old (22% of total).

Regarding education status, 86% had university education and the study result reflects more about perception (on organic food) of consumers with higher education in Sweden. The participants were also grouped according to their income level, starting from 500 € per month until >4500 € per month (see Table 2). The highest number of responses came from consumers with monthly income of >4500 € (27% of total participants) followed by consumers group with 3200–4500 € (23%).

A high number of responses came from consumers with two family member, (38%) followed by numbers of family members of 1 and 4 (19% for each group), as indicated in Table 2. About 54% of participants had no child, while about 28% have 5–14 year-old children.

Regarding means of income for participants, out of the 100 participants, about 78% have income as an employee or from their own business while about 7% are students. Only about 2% are registered as jobless while about 12% are pensioners. About 4% had no information on means of income.

### 3.2. Consumers Perception on Sustainable Food Production

The first question in the survey was: which one you think is more sustainable: organic or conventional food production?

**Figure 2:**
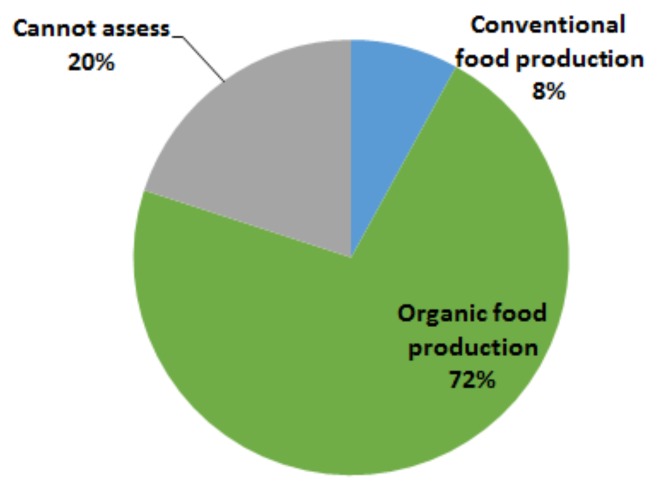
Perception of consumers on sustainable food production: in % of participants (*N* = 100).

The responses to this question have been presented in Figure 2. About 72% of participants responded that the organic food production is more sustainable than conventional methods, while about 8% said conventional farming system is more sustainable. Interestingly, about 20% refrained from deciding if organic production method is more sustainable or not and they have the following major arguments (see Table 3):

➢ Sustainability in agriculture is a vague concept and depends on geographical location of production and product type;
➢ In both organic and conventional food production approaches, sustainability could depend on how well the methods are used;
➢ At present, both organic and conventional methods are not sustainable, especially from an economic and social point of view;
➢ Sustainability is an advanced concept and more expert knowledge is needed to decide.

In order to investigate further the responses of consumers regarding the sustainability of food production methods, the following question was included: How would you reason your answer whether organic or conventional farming is more sustainable? Table 3 presents a summary of arguments by participants based on answers for the above question.

Consumers who judge conventional food production as the more sustainable system consider factors such as the increasing world population and associated food demand which needs more production per hectare of land, and the importance of genetically modified organisms (GMO) to increase food supply. The consumers in this category also believe that there is no adequate scientific evidence to say organic farming is more sustainable than conventional.

#### 3.2.1. Relation between Gender Difference and Perceptions of Sustainable Food Production System

From the gender perspective, more than male customers, the female customers perceive that organic production is more sustainable. In a previous study [20], it was indicated that women consumers tend more to purchase organic food. Similarly, Table 4 indicates that about 71% of participants who responded that organic farming was a sustainable method of food production (51 out of 72) are female participants. The relationship between gender and perception towards sustainable farming system was tested statistically (Chi-square test) with the following *null hypothesis*:

**Hypothesis** **1.** *Perception of sustainable farming methods does not depend on gender difference*.

The Pearson’s Chi-square test resulted in *p* value of 0.004, declaring that the null hypothesis should be rejected. This indicates that gender has an influence on attitudes towards sustainable food production methods and, in this case, the perception of female consumers differs significantly from that of male consumers. This indicates that female consumers could play an important role in promoting organic food production and consumption.

#### 3.2.2. Relation between Consumers’ Age and Perception of Sustainable Food Production

The participants’ age varied from 18 years to 65+ years. As indicated in Table 2, the majority (41%) of the participants were within the age range 35 to 54 years old. Table 5 describes the response of consumers from different age groups regarding sustainable food production methods. It is noticed that the number of consumers favoring organic production follows the pattern of number of participant in each age group. In order to check if the age difference has a significant impact on the perception regarding sustainable method of food production, the Chi-square test was run with the following hypothesis:

**Hypothesis** **2.** *Age difference and perceptions regarding sustainable methods of farming are independent*.

The *p* value was found to be slightly greater than 0.0503, indicating that the null hypothesis is accepted (see Table 5). This means there is no significant influence of age group difference on perceptions regarding sustainable farming method.

#### 3.2.3. Relation between Education Level and Perceptions towards Sustainable Food Production Methods

In this survey, even though the majority of participants had a university education, many participants (about 20%) found it difficult to decide between organic and conventional farming methods as the more sustainable farming method. This indicates that although many participants (72%) accept organic farming as the more sustainable method, it does not necessarily imply that participants with higher education consider organic farming as more sustainable. To check this concept statistically, Pearson’s Chi-square test was run with the hypothesis:

**Hypothesis** **3.** *Education status and perceptions regarding more sustainable farming methods are independent*.

The Chi-square test result with *p* = 0.057 highlights that the null hypothesis is acceptable (see Table 6). This indicates that there is no significant difference between participants with different education status regarding the issue of sustainable farming methods. That means that, although many participants have university education, the remaining participants without university education also mainly favored organic farming as more sustainable farming, and there are many educated people who believe organic farming may not necessarily be more sustainable than conventional.

#### 3.2.4. Relation between Household Size and Attitudes towards Sustainable Farming Methods

Table 7 depicts that many participants (27%) who judged organic farming as more sustainable method have family size of 2. However, this does not necessarily mean that participants of family size 2 always favor organic farming as the more sustainable method, because the same pattern has been noticed for groups of participants who favor conventional production or could not assess at all (see Table 7). It could be because many participants in the survey (38%) have a family size of 2. In order to increase the clarity of this observation regarding the relation between household size and attitude towards sustainable food production, Pearson’s Chi-square test was done with null hypothesis:

**Hypothesis** **4.** *Perception of consumers on sustainable food production system does not depend on household size*.

The test has resulted in *p* value of 0.86, confirming that the null hypothesis is acceptable.

#### 3.2.5. Relation between Income Level and Perceptions of Sustainable Food Production

From Table 8, about 50% of participants have an income more than 3200 € per month, while about 33% earn between 2000 and 3200 €. The remaining participants earn less than 2000 €. Although 50% of participants have an income more than 3200 €, the corresponding percentage of participants who favored organic farming method is 30%, indicating that participants with higher income level do not necessarily favor organic farming as sustainable method. This was further tested statistically with Pearson’s Chi-square test.

**Hypothesis** **5.** *Participants’ income levels and perception on sustainable farming method are independent*.

The *p* value from Pearson’s Chi-square test was 0.62, indicating that the null hypothesis is acceptable. This confirms that view of participants regarding sustainable farming method does not necessarily depend on their income level.

### 3.3. Evaluation of Food Quality

#### 3.3.1. Analyzing How Consumers Evaluate Food Quality

The consumers were asked to rank the characteristics of a product in order of importance from a list of eight parameters (characterizing food quality), indicating how the participants evaluate the food quality. The eight parameters were production in a sustainable way (not necessarily organic); Fair Trade; organic; price; appearance; nutritional values; uniformity among the products (or the pieces in the package); and regionality.

In order to investigate how consumers evaluate food quality, a question with scale of 1–8 was created:

1 = The most important characteristic to indicate product quality

8 = The least important characteristic to indicate product quality

From the survey result, there was no customer who gave rank 8 for *production in a sustainable way.* Similarly, no customer gave rank 1 and 2 for *uniformity among the products*. It has been also learnt that *regionality, production in sustainable way*, and *organic production* appeared as indicators of high quality while *uniformity among products*, *appearance,* and *price* were found to be less important characteristics as indicators of food quality.

#### 3.3.2. Relationship between Consumers’ Income Level and their Evaluation of Food Quality

**Figure 3:**
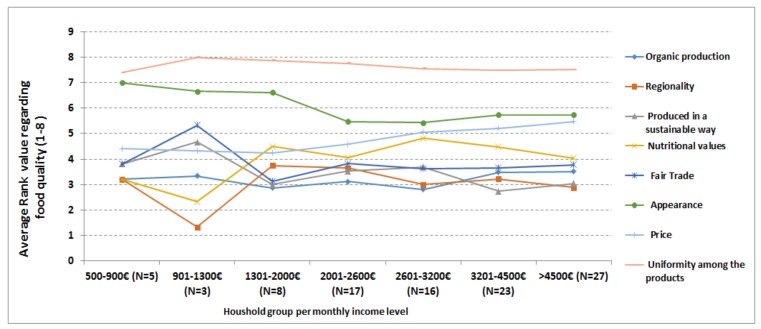
Description of food quality ranking according to income level-based household groups (*N* = 99).

The households were grouped into seven categories according to their income level and analyzed to understand how they characterize the food quality. For this, average ranking value (for example average rank value given to ‘regionality’ by participants in the 500–900 € income group) for each food quality parameter (organic production, regionality, Fair Trade etc.) were computed and presented in Figure 3. Figure 3 indicates that *regionality, organic production,* and *produced in a sustainable way* have high importance while *uniformity among products* and *appearance* have less importance in characterizing food quality by almost all household groups based on their income.

The Chi-square test was also run to understand if the food quality characterization by consumers depends on household income level or not.

**Hypothesis** **6.** Food quality characterization by consumers does not depend on their income level.

The Chi-square resulted in *p* value of 1, indicating that the characterization of food quality by consumers does not statistically depend on their income level and the null hypothesis was accepted.

### 3.4. Evaluation of Food Buying Decision

#### 3.4.1. Analyzing the Trend of Consumers’ Food Buying Decisions

The consumers were asked to rank the characteristics of a product in order of importance (in making buying decision) from a list of eight parameters (the same parameters used to characterize food quality as explained in Section 3.3). Their response indicates how the participants make food buying decision. *Production in a sustainable way, regionality, and organic production* are found to be most important characteristics, followed by Fair Trade and nutritional value. On the other hand, *uniformity among the products, appearance, and price* appeared as less important characteristics for food buying decisions. It was noticed that no participant gave the rank from 1–3 for *uniformity among products*.

#### 3.4.2. Relation between Consumers’ Income Level and Their Food Buying Decisions

**Figure 4:**
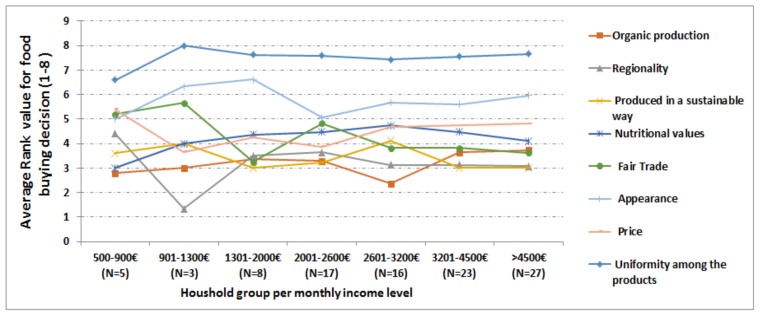
Description of food buying decision ranking according to income level based household groups (*N* = 99).

The participants’ responses were also analyzed from the income level of view to understand how they characterize their food buying decisions. In a similar way as explained in Section 3.3.2, average ranking values for each parameter (organic production, regionality, Fair Trade etc.) characterizing food buying decisions were computed and presented in Figure 4. Figure 4 depicts that *regionality, produced in a sustainable way,* and *organic production* have high importance while *uniformity among the products* and *appearance* are found to be less important food characteristics to make a buying decision. The average ranking values for food buying decisions were also used to further test statistically the relation between income level and food buying decisions.

**Hypothesis** **7.** *Consumers’ food buying decision does not depend on their income level*.

The Pearson’s Chi-square test has resulted in *p* value of 1. In this group of consumers, the result confirms that consumers’ food buying decision does not statistically depend on their income level and the null hypothesis has been accepted.

### 3.5. Relationship between Consumers Perceptions of Food Quality and Their Buying Decisions

**Figure 5:**
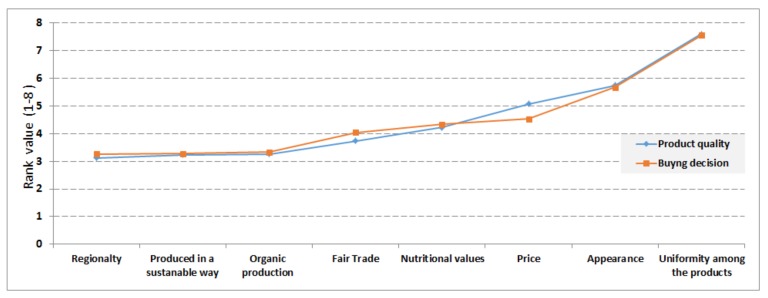
Relationship between perceived product quality and buying decisions. The average (*N* = 100) rank values (*Y*-axis) were computed for each of the eight parameters (see horizontal-axis).

Consumers’ food buying decisions could be influenced by food quality [20]. In order to test this statistically (using the current survey result), the weighted average ‘Rank values’ (1–8, and *N* = 100) have been computed for both food quality and buying decision characteristics (see Figure 5).

Then a Pearson’s correlation coefficient, *r*, was computed to test if there is a linear dependence between food quality and buying decisions of consumers. The correlation between perceptions of quality and buying decisions was found to be high with a correlation coefficient of *r* = 0.99, indicating that the buying decision and perception of participants on food quality have a strong positive correlation. Figure 5 also depicts clearly an interesting insight, that *regionality* appeared as the important parameter to characterize high quality and buying decision, followed by *produced in sustainable way* and *organic production*. Although about 72% of participants believe organic farming is more sustainable than conventional farming methods, the organic production appeared at the third level on average (following regionality and production in a sustainable way). Product *uniformity* (followed by *appearance* and *price*) appeared to be less relevant characteristics in relation to food quality and buying decisions. This indicates that there is a high demand for locally produced food, especially food produced in a sustainable way, in Sweden.

**Figure 6:**
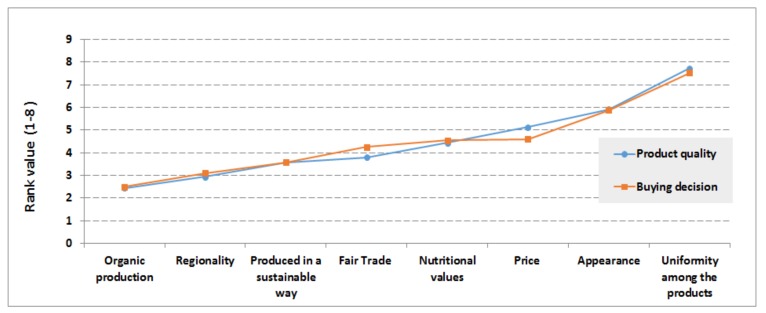
Relationship between perceived product quality and buying decision by participants who believed organic farming is more sustainable. The average (*N* = 72) rank values (vertical-axis) were computed for each of the eight parameters (see horizontal axis).

In order to understand if the participants who believed that organic farming is more sustainable have a similar food quality and buying decision relationship, weighted averages of ‘rank values’ were computed separately (i.e., *N* = 72). Figure 6 describes that *organic production, regionality,* and *production in a sustainable way* appeared as the most important characteristics for both food quality and buying decisions of consumers.

There is also a strong positive correlation between perceptions of food quality and buying decisions of this group of participants. The correlation coefficient was computed to be *r* = 0.99. It should be noticed that these group of participants (*N* = 72) put *organic production* as first criteria for food quality and buying decisions, while the overall response (*N* = 100) showed *regionality* to be first criteria as food quality and buying decisions (See Figure 5 and Figure 6). From the other end of the graphs (see Figure 5 and Figure 6), *uniformity* among products, *appearance* and *price* appeared as less important food quality and buying decision criteria in both groups of participants (i.e., *N* = 100 and *N* = 72).

It is important to note that *price* and *nutritional value* were ranked by participants to be less important in comparison to *regionality, sustainable production,* and *organic production* parameters. This indicates that consumers have a high willingness to pay more for organic food, regional food, and food produced in more sustainable way, and this supports the study results by Irandoust [19] on Swedish food consumers.

### 3.6. Purchasing Characteristics and Expenditure on Organic Food

**Figure 7:**
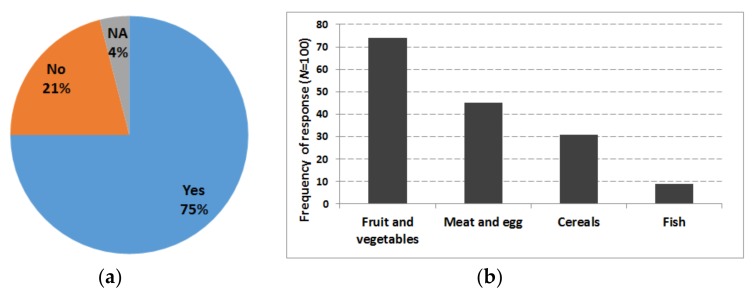
Expenditure on organic food and purchasing frequency. (**a**) Spending >14 € on organic produce per each purchase of food; (**b**) frequency of purchasing different organic items.

The participants were asked whether they expend more than 14 € on organic food per each purchase. The answer was provided as ‘*Yes*’, ‘*No*’, or ‘*NA*’ where NA = no answer (see Figure 7a). The result indicated that about 75% responded that they expend >14 € on organic produce per each food purchase.

Considering the frequency (*N* = 100) of responses regarding purchasing different organic produces (fruits and vegetables, meat and egg, cereals, and fish), organic fruits and vegetables were found to be purchased most often, followed by organic meat and egg (see Figure 7b).

### 3.7. Further Discussion and Recommendation

Organic food production method aims to enhance the sustainability of food production and supply [21]. With increasing consumer awareness of sustainable and quality food production, there is an increasing trend in organic food production and consumption [22,23]. This is because organic food production is associated with consumer health, animal welfare, food security, as well as environmental advantages [22,24]. This fact has been supported by the results of the current study, where consumers perceive the regionality of food, sustainable production, and organic production as indicators of high quality and have a willingness to pay high prices for high quality organic food.

Organic food is normally linked to sustainable food production, and it uses mainly locally available renewable resources as well as wastes and by-products of plant and animal origin [5]. As a result, organic food production and consumption are increasing in Europe. In addition, food consumption in Europe is more influenced by lifestyle and concerns for consumers’ health, environment and sustainable development [25]. Consumers in Europe are aware of the role of organic production method for sustainable development in the agriculture sector. This is supported by the results of the current study, which has indicated that the positive attitude towards sustainable food production is high and is not much influenced by consumers’ age differences. However, female consumers have a more positive attitude towards organic and sustainable food production.

In addition to food quality, organic food consumers tend to buy more from stores with organic brands that provide high-quality service [16,26]. In a study [27] on perceptions of organic food consumption in Romania, it was pointed out that a more positive consumer attitude towards organic food products could strengthen their purchasing intentions of organic food. In this regard, Swedish food retailers play an important role in promoting more environmentally sustainable food through their procurement and encouraging consumers to buy more sustainable food items [15].

In order to address the increasing demand of organic food and regional food in Sweden and the challenges accompanying this increasing trend, it is important to increase the supply of regionally produced and organic food and food raw materials as well as increasing traceability for both locally produced and imported organic products. It is important to build up more positive attitudes of consumers towards organic food via advertisements, education, and constant information flow along organic food supply chains [28].

This study was based on responses of 100 consumers. Even though the survey was open to all, the responses represent only about 0.001% of the Swedish population. Since about 96% of responses were from online survey, there is the possibility that the respondents could mostly be those who have easy access to the Internet to fill out the survey. Therefore, the results may not be necessarily representative of Swedish consumers and should be used with caution.

In order to address the increasing consumers’ demand for organic food and regional food in Sweden and to tackle challenges accompanying this increasing demand, the following recommendations have been suggested:

- Increasing the supply of regionally produced and organic food and food raw materials as well as increasing traceability for both locally produced and imported organic products is important to increase consumers’ satisfaction in Sweden.
- Promoting further study, especially targeting consumers such as the LOHAS group who are more interested in organic and regional food produced in a sustainable way. This helps to identify more specific demands and characteristics of organic food consumers with adequate scientific data, because, these group of consumers are expected to be more willing to participate in the data survey for studies to be conducted on organic food and sustainable food production.
- Further studies on consumers are needed to build a good knowledge base in relation to promoting sustainable food production. Those new studies should focus on balanced means of data collection, for instance telephone contact, face-to-face interview, and online responses.

## 4. Conclusions

This study was aimed at identifying the major behaviors and expectations of food consumers in Sweden (in relation to organic food production and consumption) and using the results to increase the satisfaction of organic food consumers by improving the value and quality of organic food production, processing, and supply. From the literature review, it was noticed that consumers’ demand for organic food products in Sweden is increasing sharply. Following this increased demand for organic food, some challenges are noticeably apparrent: difficulty in supplying adequate organic raw materials to food industry, and the risk of fraud with the food labeling system indicating the need of strong supervision of the system.

From the survey-based analysis, about 72% of total participants have the perception that organic food production method is more sustainable than conventional methods. Female consumers have strong attitudes towards organic production method. However, it was noticed that consumers (including those who have university education) might find it difficult to understand the concept of organic agriculture and to judge whether organic or conventional farming is more sustainable. Therefore, more research and education is required to generate more understanding on sustainability and organic production concepts.

Considering all participants, consumers’ judgment on food quality indicated that *regionality, sustainable way of production* and *organic production* are the most important parameters to characterize the food as high quality. On the other hand, *product uniformity, appearance,* and *price* were found to be less important parameters relatively in deciding the quality of food. The same pattern was noticed for food buying decisions, indicating that buying decision and food quality are highly related with Pearson’s correlation coefficient of *r* = 0.99. *Price* and *nutritional value* were ranked by participants to be less important in comparison to *regionality, sustainable way of production,* and *organic production* parameters. This indicates that consumers have a high willingness to pay more for organic food, regional food, and food produced in more sustainable way and this supports the study results by Irandoust (2016) on Swedish food consumers. Food retailers can use these results to increase the satisfaction of organic food consumers.

In general, the results of this study could be used by different actors along the food supply chain to improve the quality of organic food production, processing, and supply. In order to address the increasing demand of organic food in Sweden and challenges accompanying this increasing trend, it is important to increase the supply of more traceable organic food. The study results could also have input for food related policy issues.

## Figures and Tables

**Table 1:** List of null hypotheses and measurement variables.

Variables under Comparison	Null Hypothesis (Ho)
Relation between gender difference and perception of sustainable food production system	**Hypothesis 1.** *Perception of sustainable food production method does not depend on gender difference*.
Relation between consumers’ age and perception of sustainable food production	**Hypothesis 2.** *Age difference and perception regarding sustainable method of farming are independent*.
Relation between education level and perception towards sustainable food production methods	**Hypothesis 3.** *Education status and perception regarding more sustainable farming method are independent*.
Relation between household size and attitude towards sustainable farming methods	**Hypothesis 4.** *Perception of consumers of sustainable food production system does not depend on household size*.
Relation between income level and perception of sustainable food production	**Hypothesis 5.** *Participants’ income levels and perception of sustainable farming methods are independent*.
Relationship between consumers’ income level and their evaluation of food quality	**Hypothesis 6.** *Food quality characterization by consumers does not depend on their income level*.
Relation between consumers’ income level and their food buying decision	**Hypothesis 7.** *Consumers’ food buying decision does not depend on their income level*.

**Table 2:** Demographic description of participants (*N* = 100).

Demographic Characteristics	Variable	Number of Participants	Total
Gender	Male	39	100
	Female	61	
Age	18–24	4	100
	25–34	15	
	35–44	32	
	45–54	22	
	55–64	17	
	65+	10	
Education level	Primary education	1	100
	Lower secondary education	2	
	Upper secondary education	6	
	University education	86	
	Vocational education	5	
Household income (€/month)	500–900	5	100
	901–1300	3	
	1301–1500	1	
	1501–1700	0	
	1701–2000	7	
	2001–2600	17	
	2601–3200	16	
	3201–4500	23	
	≥4500	27	
	can’t answer	1	
Number of family members	1	19	100
	2	38	
	3	17	
	4	19	
	≥5	7	
Occupation	Employee or running own business	79	100
	No job (searching for job)	2	
	Student	6	
	Pensioner	12	
	other (no data)	4	
Number of children per household	No child	54	100
	<5 years old	14	
	5–14 years old	28	
	14–18 years old	4	

**Table 3:** Arguments behind the responses on perception on sustainable food production.

Perceived Sustainable Food Production	Major Arguments Indicated
Organic production	Avoiding use of chemicals, less toxins, and improving animal husbandry
	Nutrient cycles and less chemicals
	Better conditions for environment and animals
	Organic farming has environmental and ecosystem protection mindset
	Better preservation of biodiversity, small-scale farming and important cultural sites
	Organic farming ensures food for future generation i.e., Less exploitation of resources
	Organic farming requires less investment fund
	Less chemical; locally produced
	Organic farming avoids genetically modified organisim (GMOs)
	Organic products have good taste and food quality
Conventional production	Organic farming disregards importance of using GMO
	No convincing scientific reason for organic farming being better than conventional
	Organic cannot feed growing demand of increasing population
	Depending on country conventional farming can be sustainable e.g., some of participants believe that Swedish conventional farming is sustainable
	Conventional farm needs less land (and less leaking of nutrients in relation to production) than organic farming
	Conventional farming is more free from disease infestation
	There are more restrictions on organic farming. That can hinder it from being sustainable
Cannot assess (difficult to judge)	It depends on how well methods used in both cases Sustainability is a vague concept and depends on geographical location of production and product type
	More expert knowledge needed to decide
	Both organic and conventional methods are not sustainable at present e.g., From an economic and social point of view

**Table 4:** Description of gender and responses on sustainable farming method.

Gender	Organic Farming	Conventional Farming	Cannot Assess *	Total
Male	21	6	12	39
Female	51	2	8	61
Total	72	8	20	100
Chi-square test parameters	df **			*2*
	*p*			*0.004*

* ‘Cannot assess’—Some participants respond that they cannot judge which method (organic or conventional) is more sustainable; ** *df* = degree of freedom.

**Table 5:** Responses regarding sustainable food production by age group.

Age	Organic Farming	Conventional Farming	Cannot Assess	Total
18–24	4	0	0	4
25–34	11	1	3	15
35–44	23	2	7	32
45–54	18	0	4	22
55–64	12	1	4	17
65+	4	4	2	10
Total	72	8	20	100
Chi-square test parameters	*df*			*10*
	*p*			*0.0503*

**Table 6:** Relation between education level and perceptions of sustainable farming methods.

Education Status	Organic Farming	Conventional Farming	Cannot Asses	Total
Primary school education	0	1	0	1
High school education—incomplete	2	0	0	2
High school education—complete	5	0	1	6
Higher education—Vocational training	5	0	0	5
Higher education—University education	60	7	19	86
Total	72	8	20	100
Chi-square test parameters	*df*			*8*
	*p*			*0.057*

**Table 7:** Relation between household size and sustainable farming method.

Household Size	Organc Production	Conventonal Production	Cannot Asses	Total
1	13	2	4	19
2	27	4	7	38
3	14	1	3	18
4	15	0	4	19
≥5	3	1	2	6
Total	72	8	20	100
Pearson’s Chi-square test parameters	*df*			*8*
	*p*			*0.86*

**Table 8:** Income level and perceived sustainable food production method. The values are in number of participants (*N* = 100).

Perceived Sustainable Production Method	Monthly Income Range								
	500–900 €	901–1300 €	1301–2000 €	2001–2600 €	2601–3200 €	3201–4500 €	>4500 €	Cannot Decide	Total
Conventional	1	0	0	1	0	3	3	0	8
Organic	4	3	6	15	13	14	16	1	72
Do not know	0	0	2	1	3	6	8	0	20
TOTAL	5	3	8	17	16	23	27	1	100
Pearson’s Chi-Square test	*df*								*14*
	*p*								*0.62*

## References

[1] Röös E., Karlsson H. (2013). Effect of eating seasonal on the carbon footprint of Swedish vegetable consumption. J. Clean. Prod..

[2] European Parliamentary Research Service (EPRS) Short Food Supply Chains and Local Food Systems in the EU. http://www.europarl.europa.eu/RegData/etudes/BRIE/2016/586650/EPRS_BRI(2016)586650_EN.pdf.

[3] Organic Farming. https://enrd.ec.europa.eu/publications/eu-rural-review-18-organic-farming_en.

[4] Arbenz M., Gould D., Stopes C. (2016). The World of Organic Agriculture: Statistics and Emerging Trends.

[5] EC (2007). Council Regulation (EC) No. 834/2007 of 28 June 2007 on Organic Production and Labelling of Organic Products and Repealing Regulation (EEC) No. 2092/91. Off. J. Eur. Union.

[6] United Nations General Assembly (1987). Report of the World Commission on Environment and Development: Our Common Future.

[7] EISA (Undated) A Common Codex for Integrated Farming.

[8] KRAV (2016). Market Report 2016. http://www.krav.se/sites/default/files/krav_market_report_2016_eng_webb.pdf.

[9] Jönsson Å.H. (2007). Organic Apple Production in Sweden: Cultivation and Cultivars. Ph.D. Thesis.

[10] KRAV (2018). Standards for KRAV-Certified Production. http://www.krav.se/sites/default/files/krav_standards_2018.pdf.

[11] Ajzen I., Haugtvedt C.P., Herr P.M., Cardes F.R. (2008). Consumer Attitudes and Behavior. Handbook of Consumer Psychology.

[12] Billing V., Mikk M., Airi A. (2007). Local and Organic Food. EU INTERREG IIIC Project 2005–2007. http://www.interreg4c.eu/uploads/media/pdf/7_Local_and_Organic_Food_LOF.pdf.

[13] Hilmkvist N. (2015). Is Organic Greenhouse Production of Tomato in Scania feasible? Experiences and Reflections According to Swedish Growers in Scania. Master’s Thesis.

[14] Kumar B., Manrai A.K., Manrai L.A. (2017). Purchasing behavior for environmentally sustainable products: A conceptual framework and empirical study. J. Retail. Consum. Serv..

[15] Tjärnemo H., Södahl L. (2015). Swedish food retailers promoting climate smarter food choices-trapped between visions and reality?. J. Retail. Consum. Serv..

[16] Ngobo P.-V., Jean S. (2012). Does store image influence demand for organic store brands?. J. Retail. Consum. Serv..

[17] Food and Agriculture Organization of the United Nations (2001). World Markets for Organic Fruit and Vegetables: Opportunities for Developing Countries in the Production and Export of Organic Horticultural Products.

[18] Szakály Z., Popp J., Kontor E., Kovács S., Petö K., Jasák H. (2017). Attitudes of the Lifestyle of Health and Sustainability Segment in Hungary. Sustainability.

[19] Irandoust M. (2016). Modelling Consumers’ Demand for Organic Food Products: The Swedish Experience. Int. J. Food Agric. Econ..

[20] Magnusson M.K., Arova A., Hursti U.K. (2001). Attitudes towards organic foods among Swedish consumers. Br. Food J..

[21] FAO Organic Agriculture. Proceedings of the Committee on Agriculture.

[22] Shafie F.A., Rennie D. (2012). Consumer Perceptions towards Organic Food. Procedia Soc. Behav. Sci..

[23] Basha M.B., Masonb C., Shamsudin M.F., Hussain H.I., Salem M.A. (2015). Consumers Attitude towards Organic Food. Procedia Econ. Financ..

[24] Brul P., Mattsson E., Parrott N., Stopes C. (2013). Organic Food and Farming for All.

[25] Oroian C.F., Safirescu C.O., Harun R., Chiciudean G.O., Arion F.H., Muresan I.C., Bordeanu B.M. (2017). Consumers’ Attitudes towards Organic Products and Sustainable Development: A Case Study of Romania. Sustainability.

[26] Mutlu N. (2007). Consumer Attitude and Behavior towards Organic Food: Cross-Cultural Study of Turkey and Germany. Master’s Thesis.

[27] Petrescu A.G., Oncioiu I., Petrescu M. (2017). Perception of Organic Food Consumption in Romania. Foods.

[28] Stolz H., Stolze M., Hamm U., Janssen M., Ruto E. (2011). Consumer attitudes towards organic versus conventional food with specific quality attributes. NJAS Wagening. J. Life Sci..

